# Successful treatment of pulmonary hypertension due to HIV infection and amphetamine snorting: a case report

**DOI:** 10.3389/fmed.2025.1556041

**Published:** 2025-04-23

**Authors:** Cuiming Sun, Ying Wen, Yanli Chen

**Affiliations:** ^1^Second Department of Infectious Diseases, The First Hospital of China Medical University, Shenyang, China; ^2^Department of Cardiology, The First Hospital of China Medical University, Shenyang, China

**Keywords:** pulmonary hypertension, HIV infection, amphetamine, sildenafil, anti-retroviral therapy

## Abstract

HIV infection is one of the high-risk factors for pulmonary hypertension (PH). HIV-related PH is associated with non-AIDS-related comorbidities. In this study, we report a case of pulmonary hypertension with several concomitant risk factors who experienced a complete clinical improvement after drug rehabilitation, application of anti-retroviral therapy (ART), and sildenafil. A 37-year-old HIV-positive man complained about worsening dyspnea was diagnosed with severe PH. PH in this case was characterized by association with multi-factors, including amphetamine inhalation, HIV infection, bacterial liver abscess, history of splenectomy, and past ventricular septal defect, which was different from previously reported HIV-related PH cases who were associated solely with HIV infection. Our case intends to raise awareness that PH should be suspected among HIV-positive patients with unexplained dyspnea. It is notable that investigating the coexistent risk factors and a multidisciplinary team are crucial for early diagnosis and better prognosis of HIV-related PH.

## 1 Introduction

Pulmonary hypertension (PH) is uncommon in the HIV-positive population; however, it could be a cause of mortality associated with non-acquired immune deficiency syndrome (AIDS). The prevalence of PH is higher in HIV-infected patients than in the general population (1). PH is associated with various comorbidities, including chronic obstructive pulmonary disease, left heart disease, chronic thromboembolic pulmonary disease, connective tissue disease, and co-infections (2, 3). PH should be considered in HIV-infected patients with progressive dyspnea and pedal edema. Hemodynamics, respiratory, and cardiac functions should be further accessed. In this study, we report a case of PH with several concomitant risk factors who experienced a complete clinical recovery after drug rehabilitation, application of sildenafil, and anti-retroviral therapy (ART) initiation.

## 2 Case presentation

### 2.1 Past history

We report a case of HIV-positive 41-year-old Chinese man who suffered from PH with several concomitant risk factors in August 2017. He had a history of ventricular septal defect (VSD), which was surgically repaired at the age of 14 years, and a history of trauma and splenectomy at the age of 20 years. He was diagnosed with HIV-1 infection in September 2013 and developed severe liver abscess in November 2013. In November 2013, his CD4^+^ T-cell count was 1,428/μL, and he declined ART. Echocardiography showed no left and right ventricular shunt, mild mitral valve, and tricuspid regurgitation. The blood flow velocity of the mitral, tricuspid, aortic, and pulmonary valves was normal. Cardiac function was normal under quiescent conditions. With 2 months of therapy, including carbapenem, ultrasound-guided per-cutaneous needle aspiration, and catheter drainage, the liver abscess was cured. He had a history of drug abuse since recovery from liver abscess. He inhaled amphetamine more and more frequently since 2015.

### 2.2 Present history

**Figure 1:**
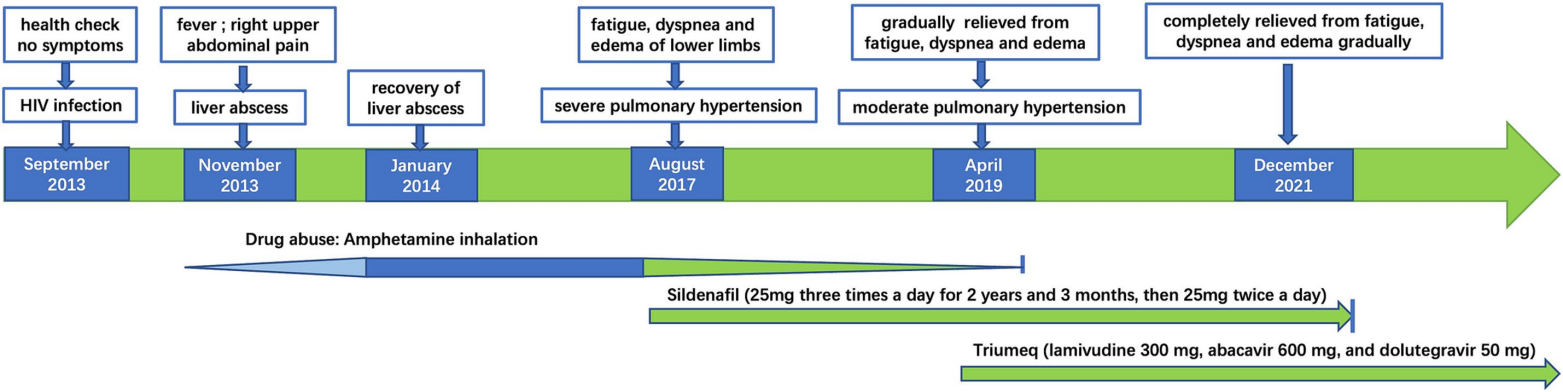
Summary of the treatments received and clinical manifestation.

**Figure 2:**
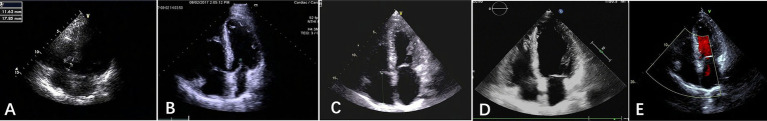
Apical four-chamber echocardiograms from January 2014 to December 2020. **(A)** January 2014; **(B)**. August 2017; **(C)**. April 2019; **(D)**. August 2019; **(E)**. December 2020. The right ventricle was enlarged in 2017 **(B)** and returned to nearly normal in 2019 **(D)** and 2020 **(E)**.

In August 2017, he reported severe fatigue, worsening dyspnea, and edema of the bilateral lower extremity for a month. His blood pressure was 130/83 mmHg, and blood oxygen saturation was 91% on room air. The N-terminal pro-B-type natriuretic peptide (NT-Pro-BNP) was 3,554 pg./mL. The chest CT scan showed an increase in pulmonary vessel diameter. Echocardiography showed mild tricuspid regurgitation with a reflux velocity of 4.6 m/s and an estimated pulmonary artery pressure of 97 mmHg. He was diagnosed with PH. To accurate evaluate the pulmonary artery pressure (PAP) and stratify PH, right heart catheterization (RHC) was recommended, but he refused due to its invasive nature. The distance of his 6-min walking test was approximately 270 m, and his cardiac function was grade III according to the World Health Organization (WHO) classification (4). A combination of endothelin receptor antagonist (ERA) (bosentan) and phosphodiesterase type 5 inhibitor (PDE5I) (sildenafil) was prescribed, but he only agreed to adopt monotherapy with sildenafil citrate (25 mg, three times a day) due to the high price of bosentan. His HIV viral load was 4,220 copies/ml (COBAS TaqMan V2.0 RT-PCR, Roche; detection limit of 20 copies/ml) with the CD4^+^ T-cell count of 806/μL, while he still declined ART. The symptoms of dyspnea, fatigue, and edema were relieved gradually by undergoing sildenafil monotherapy without diuretic application. Hepatic and renal functions were monitored during sildenafil therapy. The levels of serum transaminase, bilirubin, and creatinine remained within normal range. Because of the possible relationship between PH and amphetamine abuse (5), drug rehabilitation was strongly recommended. With psychosocial help, he reduced the frequency of amphetamine inhalation and was rehabilitated completely in January 2019. In April 2019, his echocardiography revealed that the reflux velocity of the tricuspid was 3.8 m/s, with an estimated pulmonary artery pressure of 68 mmHg. His plasma HIV viral load was 28,000 copies/mL, and he initiated ART (Triumeq one tablet per day, including lamivudine 300 mg, abacavir 600 mg, and dolutegravir 50 mg). The HIV viral load decreased to less than 20 copies/ml half a year after ART. Then, he tapered sildenafil usage (25 mg, twice daily) since November 2019. In December 2021, he was relieved of fatigue, dyspnea, and pedal edema. His physical capacity was improved, as evidenced by an increase in the distance covered during the 6-min walking test to 560 m and a decrease in NT-Pro-BNP decreased to 75 pg./mL. His cardiac function was grade I according to the WHO classification. The arterial oxygen saturation was 96% in blood gas analysis performed on room air. Cardiac echo showed that the reflux velocity of the tricuspid decreased to 2.6 m/s with the estimated pulmonary artery pressure of 27 mmHg. He returned to normal work and daily life and withdrew from sildenafil subsequently. He was without significant complaints at follow-up telemedicine visits. We summarized the treatments received and clinical manifestation in Figure 1, images of the echocardiogram in Figure 2, and basic clinical characteristics in Table 1.

**Table 1:** Baseline characteristics of the included time point.

Clinical Findings	2013.11	2014.01	2017.08	2019.04	2021.12
Estimated PAP (mmHg)	-	Normal	97	68	27
WHO functional classification of PH	I	I	III	II	I
RVID (mm)	-	25	36	27	16
LVDD (mm)	-	50	47	54	54
EF (%)	-	58	60	60	60
Velocity of tricuspid regurgitation (m/s)	-	-	4.6	3.8	2.6
HIV viral load (copies/ml)	-	5,820	4,220	28,000	<20
CD4^+^ T cell count (cells/μl)	1,428	1,337	806	3276	1,232
Platelet count (×10^9^)	670	819	-	408	443
NT-Pro-BNP (pg/mL)	-	-	3554	-	75
6MWT (meter)	-	-	270	-	560

PAP, pulmonary artery pressure; PH, pulmonary hypertension; RVID, right ventricular internal diameter; LVDD, left ventricular end-diastolic diameter.

## 3 Discussion

PH in this case was characterized by association with multi-factors, including amphetamine inhalation, HIV infection, the history of splenectomy, and past VSD, which was different from previously reported cases with HIV-related PH who were mainly associated solely with HIV infection (6). First, drug abuse (cocaine, amphetamine, or amphetamine derivatives such as methamphetamine) is commonly found among patients with HIV infection (7). Amphetamine is considered a possible risk factor for PH. However, there has been no study providing precise epidemiologic data on amphetamine-associated PH. It induces DNA damage in pulmonary artery endothelial cells through oxidative stress, promotes pulmonary vasoconstriction, and induces reactive oxygen species (ROS) production by activating nicotinamide adenine dinucleotide phosphate oxidase (8). The right ventricle end-diastolic volume disorder, tricuspid regurgitation, and PH were also observed among patients with a history of amphetamine abuse (6). Genetic susceptibility, such as BMPR2 mutations, may synergize with amphetamine-induced damage, accelerating PH progression (9). Second, before and at the time of PH diagnosis, CD4^+^ T-cell counts in this case were always within the normal range. However, his immune function might be overestimated by CD4 lymphocytes due to asplenia (10). HIV-PH occurs in all stages of HIV infection, which is irrelative to the status of immune deficiency and CD4^+^ T-cell counts (11). Nef, gp120, and Tat proteins are key players in triggering vascular endothelial injury and the development of HIV-PH (12). Drug abuse of amphetamine acts as a second hit to these proteins. Third, splenectomy increases the production of anion phospholipids and platelet-derived microparticles, which act as procoagulants and contribute to thrombus formation (13, 14). Due to the absence of splenic filtration, abnormal red blood cells are retained in the peripheral circulation, thereby activating the coagulating proteases. Thus, splenectomy is considered a risk factor for chronic thromboembolic pulmonary hypertension (CTEPH). For patients undergoing splenectomy who present with dyspnea, pulmonary arterial pressure should be evaluated by echocardiography (15). CTEPH for this case could be excluded as evidence of pulmonary artery thromboembolism was lacking, as evaluated by CT pulmonary angiogram. Fourth, liver abscesses and VSD could cause cardiac overload, which is related to the pathogenesis of PH. Concurrent systemic inflammation and oxidative stress during liver abscesses exacerbate pulmonary vasoconstriction and fibrosis (16). In VSD, chronic left-to-right shunting leads to volume and pressure overload in the pulmonary vasculature, triggering endothelial injury and vascular remodeling. This process can ultimately result in Eisenmenger syndrome, which may further exacerbate PH. However, symptoms of PH, including dyspnea, did not appear when liver abscess was diagnosed. VSD was also surgically repaired when the patient was 14 years old. Thus, liver abscess and VSD do not have a cause–effect relationship with the occurrence of PH in this case. In addition, there were no signs of chronic liver disease or portal hypertension. Thus, PH related to portal hypertension was excluded.

Compared to idiopathic pulmonary artery hypertension (IPAH) patients, HIV-PH patients have higher rates of comorbidities, poorer treatment adherence, and a greater likelihood of methamphetamine. In addition to the combination of ERA and PDE5I for methamphetamine-associated PH, prostacyclin analogs are also recommended for patients in high-risk groups. Until recently, approximately half of the patients with methamphetamine-associated PH had received dual combination therapy (17). In one study, 60.3% methamphetamine-associated PH patients were treated with a combination therapy including prostacyclin analogs. Their 5-year survival rate was 47.2%, which was worse than 64.5% in IPAH patients (18). Currently, there are no treatment guidelines specific to amphetamine-associated PH patients or HIV-PH patients. Increasing evidence supports the finding that ART, bosentan, and prostaglandin are beneficial in improving hemodynamic and functional status in HIV-related PH, while PDE5I is only an option for patients who do not tolerate bosentan (19). Importantly, in a recent real-world study with oral monotherapy as the main first-line treatment, the 5-year survival rate was 74.0% in HIV-PH, showing similar efficacy to that of patients with IPAH/familial pulmonary artery hypertension (FPAH) (1), which inspired clinicians to further carry out a large sample study. Moreover, recent studies also indicated a more favorable effect of PDE5I used as mono-or add-on therapy in HIV-PAH than IPAH (20). Notably, this case showed a favorable response to sildenafil monotherapy, which demonstrated similar efficacy to previous reports of HIV-PH patients (21, 22). Sildenafil has a high concentration in lung tissue, and it reduces pulmonary vascular pressure by inhibiting PDE. An *in vitro* study demonstrated that sildenafil led to the relaxation of smooth muscle via the upregulated production of cyclic guanosine 5-monophosphate dependent on nitric oxide (23). To date, no trials have become available to evaluate the still controversial effect of ART on the progression of HIV-PAH. Although ART does not appear to decrease the occurrence of PH, it may slow the progress behind the development of HIV-PH by inhibiting HIV replication and suppressing abnormal T-cell activation. ART also prevents opportunistic infections, such as tuberculosis infection, which could exacerbate PH. Thus, ART should be recommended to all HIV-positive PH patients, irrespective of their CD4^+^ T-cell counts (11).

The long-term follow-up of this HIV-PH patient suggests that PH screening and cardiac function evaluation are important for HIV-infected patients, especially for drug addicts with complaints of dyspnea. However, there are several limitations to this study. First, PH is defined as a mean PAP of >20 mmHg, as measured by RHC. Until recently, only a few small sample clinical studies had conducted RHC among HIV-positive patients (24), highlighting a multifactorial societal and economic challenge. We should further focus on developing non-invasive modalities, including echocardiography, cardiac magnetic resonance (CMR), and emerging markers such as growth differentiation factor-15 (GDF-15) for diagnosis, risk assessment, and prediction, especially in the HIV-positive PH population. Integrated multi-modal approaches (echocardiography + CMR + biomarkers) enhance diagnostic accuracy, although RHC remains essential for definitive hemodynamic classification and targeted therapy initiation. Advances in artificial intelligence (AI)-driven imaging analysis and circulating microRNAs hold promise for non-invasive stratification in future. Second, some of the clinical data, such as the information about the VSD surgery, were missing due to its remote history. V/Q scan and pro-B-type natriuretic peptide were not monitored regularly, highlighting the importance of multidisciplinary teamwork with HIV specialists, cardiologists, imaging experts, and pharmacologists.

## 4 Conclusion

In conclusion, regular screening of echocardiography should be primarily recommended for HIV-positive patients with unexplained dyspnea (2), especially those with a history of PH-associated drug abuse (such as amphetamine use). Then, the N-terminal pro-B-type natriuretic peptide, 6-min walking test, WHO functional classification, and right heart catheterization could be further warranted for risk stratification of PH. The high-risk factors for PH should be addressed as early as possible, including drug rehabilitation. HIV-PH patients without definite coexistent risk factors should receive individualized PH treatment, according to the guidelines. Meanwhile, ART remains vital for HIV-PH therapy. Importantly, a multidisciplinary team is crucial for early diagnosis and improved prognosis of HIV-PH.

## Data Availability

The original contributions presented in the study are included in the article/supplementary material, further inquiries can be directed to the corresponding authors.
